# Hemodynamic and Biologic Determinates of Arteriovenous Fistula Outcomes in Renal Failure Patients

**DOI:** 10.1155/2015/171674

**Published:** 2015-10-01

**Authors:** Mary Hammes

**Affiliations:** Department of Medicine, Nephrology Section, The University of Chicago, 5841 South Maryland Avenue, MC5100, Chicago, IL 60637, USA

## Abstract

The outcome of patients with end-stage renal disease on hemodialysis depends on a functioning vascular access. Although a variety of access options are available, the arteriovenous fistula remains the best vascular access. Unfortunately the success rate of mature fistula use remains poor. The creation of an arteriovenous fistula is followed by altered hemodynamic and biological changes that may result in neointimal hyperplasia and eventual venous stenosis. This review provides an overview of these changes and the needed research to provide a long lasting vascular access and hence improve outcomes for patients with end-stage renal disease.

## 1. Introduction

End-stage renal disease (ESRD) affects an increasing number of patients each year with a staggering estimate of almost 640,000 patients receiving dialysis at the end of 2012 in the United States [1]. The primary cause of ESRD is diabetes followed by hypertension [1]. Patients with ESRD have a high prevalence of concomitant cardiovascular disease, which is the primary cause of morbidity and mortality in this patient population [1]. The most common modality chosen for renal replacement therapy is hemodialysis, for which a vascular access is required. The vascular access choice influences and contributes to the overall morbidity and mortality of the patient [2, 3].

Hemodialysis vascular access type includes the preferred arteriovenous fistula (AVF), the arteriovenous graft followed by a central venous catheter [1, 2]. The best access to place with least complications is the AVF. When an AVF is placed, an artery is anastomosed to a vein and over a period of 2-3 months the vein becomes “arterialized,” a process that is necessary prior to use of hemodialysis [4]. The first access recommended is the lower arm radiocephalic fistula (RCF) although these commonly fail especially in the elderly and those with diabetes [5, 6]. The second preferred site for an AVF is the brachiocephalic (BCF) which are being placed at an increased number. The third fistula configuration recommended is a brachiobasilic (BBF). The problem is that many of these fistulas fail for unknown reasons. One-year patency rates range from 60 to 65% [7, 8], with 60% of fistulas not suitable for dialysis between 4 and 5 months after surgery [9]. Medical management with antiplatelet agents such as ASA and Clopidogrel have failed to make a difference [10, 11]. This is likely due to the fact that these agents do not address the primary cause of access failure, neointimal hyperplasia (NH) leading to venous stenosis.

Once venous stenosis occurs with clinical symptoms such as painful swelling of an extremity, skin ulceration, venous hypertension, or subsequent poor function of the access, treatment options include angioplasty, stent, or surgical revision [12]. The treatment is dependent on the specific site, characteristics, and hemodynamics of the lesion [12]. For example, the most common location for stenosis in a RCF is near the anastomosis, while cephalic arch stenosis is frequently encountered in BCF [12, 13]. Cephalic arch stenosis is often treated with repeat angioplasty and stenting until fistula failure occurs [13].

Venous stenosis as a result of NH is poorly understood [14, 15]. There are multiple factors which influence the outcome of an AVF including demographics, adjuvant therapies, underlying histology, cytokines, oxidative stress, and hemodynamics [16, 17]. There are few trials which look at the biology of why a fistula fails or address treatment options in prospective trials. This review highlights known hemodynamic and biologic determinates of fistula failure and suggest research areas which need to be explored.

## 2. Hemodynamics of an Arteriovenous Fistula (AVF)

**Figure 1:**
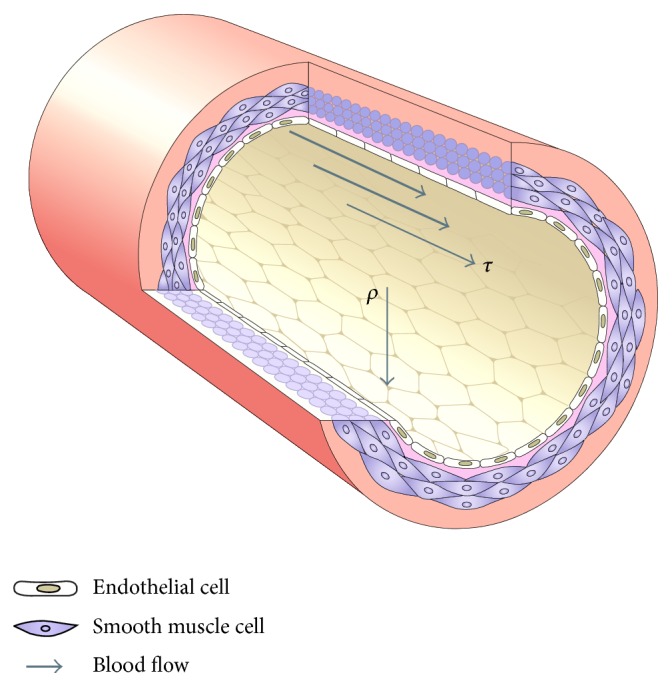
Schematic of a vessel. The white layer shows smooth endothelial cells; the purple layer smooth muscle cells; *ρ* shows direction of pressure; *τ* shows direction of wall shear stress. Figure reprinted by permission from Macmillian Publishers Ltd.: Nature Reviews Molecular Cell Biology, 10, 2009.

Creation of an AVF requires a surgical anastomosis of a high pressure artery to a low pressure vein which causes an increase in wall shear stress (WSS) and tension. The pressure increase in the venous outflow tract will lead to medial thickening, the definition of venous arterialization. Pressure is defined as the perpendicular force (*ρ*) exerted in a vessel, whereas the WSS is the parallel force (*τ*) (Figure 1) [18]. Normally the luminal diameter will increase in an attempt to reduce the WSS back to pre-AVF levels. The next result is a dilated vein with a thickened media, the perfect vessel for a fistula suitable for use for hemodialysis. Corpataux et al. [19] summarized this phenomenon in a study where hemodynamic changes were demonstrated in six patients with a lower arm AVF using an ultrasound Doppler device. Within the first week after fistula creation, the blood flow and WSS increased substantially in the vein. The increased flow resulted in a venous luminal diameter increase, a process necessary for cannulation. The WSS gradually returned back to normal by 12 weeks. In this study, findings were also apparent at the arterial side, though to a lesser extent [19].

**Figure 2:**
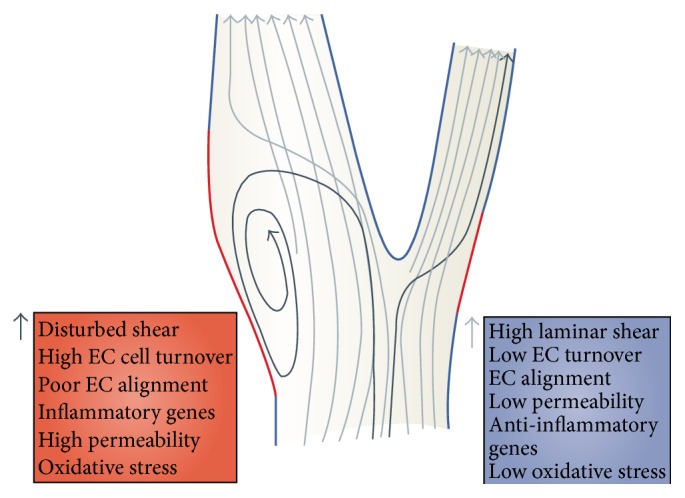
Schematic of a vessel with disturbed shear flow on the left and laminar flow on the right. Figure reprinted by permission from Macmillian Publishers Ltd.: Nature Reviews Molecular Cell Biology, 10, 2009.

Problems arise in vasculature physics when a bend or curve happens which is frequently the case especially when an AVF is being constructed. Normal flow through a straight vessel is smooth and laminar as shown by the vessel on the right side of Figure 2 [18]. The endothelial cells in this instance are at steady state, with low cell turnover, low permeability, and low level of anti-inflammatory genes and oxidative stress. The area of abnormal WSS (red) is minimal. However, when a bend or curve arises laminar flow becomes turbulent as shown in the left side of Figure 2 [18]. With turbulent flow the endothelial cell turnover is high with poor alignment, inflammatory genes activation, and increase in oxidative stress. The area of abnormal WSS is much larger. Abnormal turbulent flow causes low WSS, denuding endothelial cells, excitation of pathways which eventually lead to NH [16]. Jia et al. have recently shown in study of AVF creation in canines that NH has a strong inverse correlation with WSS levels and also is related to flow patterns [20].

## 3. Underlying Histology and Progression to Neointimal Hyperplasia

**Figure 3:**
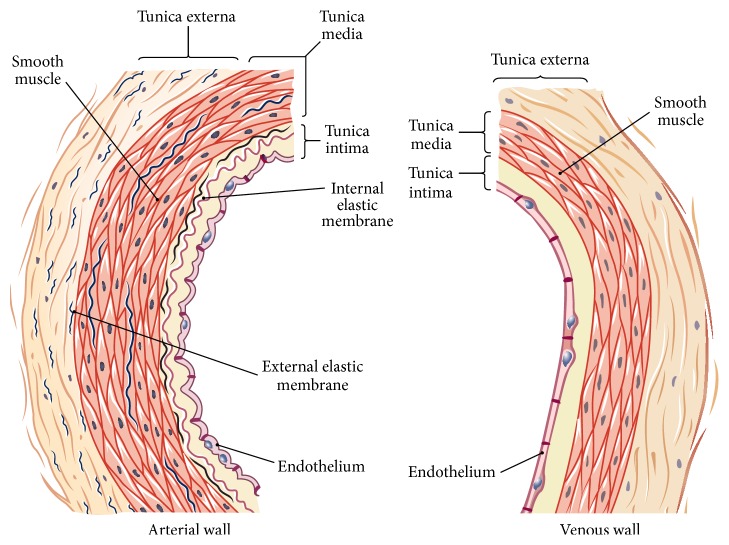
Schematic of an arterial wall on the left and venous wall on the right. Note increased tunica media in an arterial wall.

The basic histology of an artery and a vein is very different (Figure 3) [21]. A normal artery has a smooth endothelial cell lining with the tunica intima defined as the boundary of the endothelial cell to the elastic lamina. The tunica media in an artery is normally much thicker than a vein with an increased amount of elastin [21]. When a fistula is created for hemodialysis, the patient often had chronic renal failure for several years, which causes underlying changes in the vein and artery including increased arterial and venous calcification [22] and NH [23]. When a fistula is then created, the changes in WSS and pressure sensed by the endothelial cells signal vasodilating agents, such as nitric oxide (NO), growth factors that control vascular smooth muscle cell (VSMC) migration and proliferation, and cellular adhesion molecules [17]. Upregulation of proteases such as matrix metalloproteinase and cathepsins result in matrix degradation and restructuring of the luminal expansion [17]. Little is known about the necessary outward remodeling of VSMC, although this is highly important [17]. In summary, the underlying histology of the vessels used to create an AVF, specifically calcification, elastin, and collagen deposition, predicts the ability of a vein to dilate after fistula creation.

The morphometric and histologic characteristics of the veins used for fistula creation have been studied. Lazich et al. have shown that the vein used to create BCF is larger in diabetics when compared to nondiabetics [24]. In particular the internal lumen and intimal and medial area were all found to be more dilated in diabetics [24]. This altered remodeling may be beneficial as previous reports have shown that cephalic arch venous stenosis is attenuated in diabetics with BCF [25–27]. Vascular wall remodeling differs between diabetics and nondiabetics with increased NH in the former [23, 24]. As NH progresses, this can dramatically decrease the lumen size and lead to negative vascular remodeling and vasoconstriction. All components of the vein including the adventitia are now recognized as contributing to the process of NH after fistula construction [8]. Most past research focuses on NH although outward remodeling is an equally important process that could preserve the vein lumen and predict the outcome of the AVF over time [8]. This critical balance between NH and outward remodeling in a vein when a fistula is created requires further exploration.

## 4. Nitric Oxide (NO), Asymmetric Dimethylarginine (ADMA), and Vasodilator Effects

**Figure 4:**
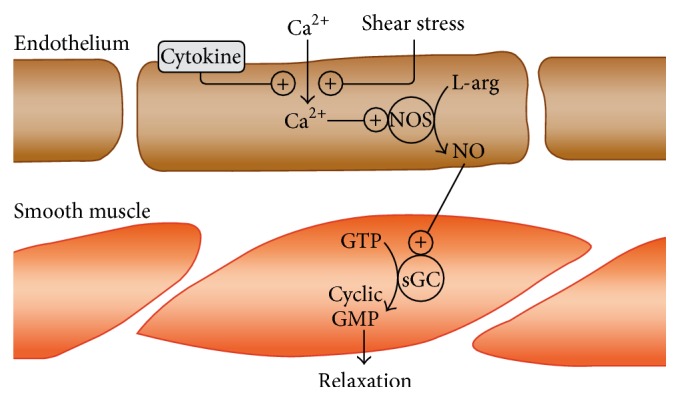
Schematic of vascular endothelium. Shear stress induces calcium dependent activation of nitric oxide synthetase resulting in smooth muscle relaxation. Figure reprinted by permission from Molecular Diversity Presevation International: International Journal Molecular Science, 13, 2012.

Fistula creation causes a passive vascular distension and a dramatic release of NO from the endothelial cells [28]. The turbulent flow induced by creation of a fistula causes an increased global shear stress which increases nitric oxide synthetase converting L-arginine to NO (Figure 4) [28]. The NO activates soluble guanylyl cyclase (sGC) to cause GTP to be converted to cyclic guanosine monophosphate cyclic GMP causing smooth muscle cell relaxation. There is a delicate balance between endothelial cell activation which is needed for vein dilation and endothelial cell dysfunction [29]. Endothelial cell dysfunction could result from increased oxidative stress and lead to vasoconstriction and smooth muscle cell proliferation which could result in NH and contribute over time to access failure.

A contributing factor to endothelial cell dysfunction is a dramatic elevation of asymmetric dimethylarginine (ADMA) known to be increased in patients with renal failure [30]. ADMA, a metabolic by-product of protein metabolism inhibits the conversion of L-arginine to NO, reducing vascular compliance, increasing vascular resistance, and limiting blood flow [30]. Hammes et al. showed a 10-fold increase in ADMA in patients with ESRD when compared to patients without renal failure [31]. The procedure of hemodialysis itself is also associated with an even greater rise in ADMA [31]. Efforts to understand the complex pathway that leads to devastating ESRD and corresponding vascular disease have identified an accelerated calcific process, observed even in small vessels. Elevated ADMA is a step in the pathway of vascular disease in renal failure as it has been found to contribute to CKD progression with an associated increase in transforming growth factor TGF-*β*1 and subsequent vascular collagen deposition. [32]. The biologic effects of ADMA on AVF contribute to venous stenosis [33] and are the subject of future research investigation.

The vascular endothelium in an artery and vein respond differently to blood flow, especially if there is associated atherosclerosis [34]. The release of eNOS and resultant NO is necessary for adequate vein dilation, a part of arterialization. Endothelial cells in arteries and veins are structurally and functionally different including the ability to adjust to changes in shear stress and release of eNOS [35]. There are many inhibitors of NO, of most interest are Rho-kinases (ROCKs). ROCKs are small guanosine triphosphate binding proteins which mediate smooth muscle contraction, cell migration, and proliferation by inhibition of eNOS [36]. Inhibition of ROCKs has been shown to decrease NH [37]. Molecular events which cause NO release are under the control of ROCKs and warrant exploration in an AVF model.

## 5. Cytokines/Inflammation

Inflammation is a primary stimulus for NH. There is marked upregulation in proinflammatory genes and progressive neointimal formation in the venous vasculature in an AVF which contributes to the aggressive venous stenosis observed. Nath et al. [38] has shown an upregulation of genes including TGF-*β*1 which, in the venous vasculature in the AVF model in the rat, are accompanied by intimal hyperplasia. NH occurred in variable degrees by 5 weeks after establishing a fistula, and by 16 weeks, such NH was progressive and pronounced with abundant extracellular matrix. In human subjects, levels of inflammatory biomarkers have been harvested in surgically thrombosed fistulae. Pronounced intimal thickening in stenosed fistulas was associated with expression of TGF-*β*1 and insulin-like growth factor when compared to controls [39].

Research in coronary artery bypass grafts has given clues as to the mechanism of venous stenosis in arterialized veins used for hemodialysis. Graft failure is common following coronary artery bypass grafting [40, 41] puzzling vascular biologists and surgeons as to mechanisms. Yuan et al. [42] have shown that severe vascular wall degeneration and collagen deposition together with overexpressed TGF-*β* signaling cytokines were responsible for failure (early and late) of the saphenous vein and radial artery grafts. As TGF-*β* is responsible for NH in a number of vascular disease models that have similarities to the arterialized vein in an AVF, targets to impede this early gene signaling may be future directions to help retard the aggressive NH which occurs in AVF.

## 6. Oxidative Stress of Dialysis

Patients with renal failure have several risk factors which predispose them to oxidative stress, including age, and underlying disease including diabetes and hypertension. Hemodialysis is a treatment which contributes to this oxidative stress by evoking a dramatic change in the blood flow through a fistula. The continuous volume and pressure changes as a result of intradialytic volume gains can cause significant physiologic stress on the endothelium of a vein. Moreover the hemodialysis treatment itself has been shown to shear endothelial cells and reduce nitric oxide formation [43]. When an access is considered “mature” and cannulation begins, this could also worsen the oxidative stress contributing to NH [44, 45].

Oxidative stress has been linked to atherosclerosis by contributing to endothelial dysfunction and intima-media thickness. Weiss et al. [46] used markers of oxidative stress to study 11 AVF and 15 AVGs at the time of surgical resection or revision. Markers of cell growth and proliferation were endothelin-1 (ET-1), a potent mutagenic peptide implicated in the formation of intimal hyperplasia: TGF-*β*, a stimulus to vascular cell growth and matrix production and platelet-derived growth factor (PDGF), a mediator of intimal hyperplasia. All specimens studied showed significant NH. The neointima close to the vascular lumen of the AVF and the pseudointima close to the lumen of the ePTFE graft were positive for oxidative stress markers. At sites of injury, as evident by histological inflammation and healing, expression of oxidative markers was more intense. These findings support intimal injury and resultant oxidative stress as a direct result of fistula construction and cannulation contributing to NH.

## 7. Future Directions

Intensive research to determine the early events that trigger NH in an arterialized vein is needed as the process of NH starts when the fistula is created [17]. The optimal mismatch of shear stress and pressure in both the vein and artery are necessary to allow for some medial thickness without aggressive NH setting in. Anastomotic design and strategies and devices to define optimal WSS are in the process of being developed [47]. Computational models have been developed and are able to predict the clinical relevance of WSS in predicting AVF maturation and venous stenosis [48–50]. Randomized clinical trials are needed to determine the utility of CFD to improve AVF outcomes. NO production and VSMC reorganization in outward remodeling in AVF likely play a role in the ability of a vein to mature to support dialysis and are targets of future research. Early vascular biological events need to be unraveled.

The current approach to placement of a vascular access should be revisited. The goal of preoperative evaluation is to provide a well-functioning access for a patient that will last a life span of a patient with ESRD. Current work-up includes physical exam, preoperative color duplex Doppler ultrasound, and/or venogram to determine suitable arterial/vein diameters and adequate blood flow. Assessment of Doppler ultrasonographic assessment of flow-mediated dilatation has been used to assess preoperative vascular health but has not been found to predict fistula success [51]. The diameter of the vein has been shown to correlate with successful outcome in some but not all studies [52]. The intraluminal area and virtual histology available by such tools as intravascular ultrasound enable a more indebt assessment of endothelium [53]. This diagnostic procedure may provide intraluminal images, allowing for more precise assessment of veins, suitable and adequate for vein maturation, than external luminal diameters provided by traditional methods. A multifactorial approach evaluating arterial and venous function is necessary to predict AVF success.

The definition of a mature AFV is vague. Current guidelines define a mature access as one that has a blood flow of at least 600 mL/min and is 6 mm in diameter and less than 6 mm below the skin surface. In clinical practice, these parameters cannot be reliably measured in an outpatient setting. Recent investigation has defined the type of blood flow in a fistula as an important determinate in maturation [54]. Cannulation techniques and skills must improve. Continued education in the anatomy of the AVF and physical assessment is crucial. New techniques for cannulation of difficult or deep veins are being developed [55]. The rapid blood flow with hemodialysis causes excessive turbulence and may not be optimal to the endothelium of the arterialized vein, predisposing to NH. We need to decrease inflammation as much as possible with each dialysis treatment. Surveillance to detect access dysfunction including high flow states needs to be refined [56]. In summary, we must continue to investigate the hemodynamics and vascular biology of the AVF and develop better clinical parameters that confirm adequate AVF function if the outcomes are going to improve.
